# Supplementary material for: Effects of Coronavirus Persistence on the Genome Structure and Subsequent Gene Expression, Pathogenicity and Adaptation Capability
Source: Cells. 2020 Oct 19;9(10):2322. doi: 10.3390/cells9102322 (PMC7589090; doi:10.3390/cells9102322)
Supplement: Supplementary file 1 [file cells-09-02322-s001.pdf]

Supplementary Materials

Effects of coronavirus persistence on the genome structure and subsequent gene expression, pathogenicity and adaptation capability

Ching-Hung Lin[1], Cheng-Yao Yang[1], Meilin Wang[2], Shan-Chia Ou[3], Chen-Yu Lo[1], Tsung-Lin Tsai[1], Hung-Yi Wu[1]*

1 Graduate Institute of Veterinary Pathobiology, College of Veterinary Medicine, National Chung Hsing University, Taichung 40227, Taiwan; tw23whale@hotmail.com (C.-H.L.); yangchengyao@nchu.edu.tw (C.-Y. Y); axfbji7917@gmail.com (C.-Y.L.); windtaker10@msn.com (T.-L.T.)

2 Department of Microbiology and Immunology, School of Medicine, Chung-Shan Medical University and Clinical Laboratory, Chung-Shan Medical University Hospital, Taichung 40201, Taiwan ; wml@csmu.edu.tw (M. W.)

3 Graduate Institute of Microbiology and Public Health, College of Veterinary Medicine, National Chung Hsing University, Taichung 40227, Taiwan; scou@dragon.nchu.edu.tw (S.-C.O.)

* Correspondence; E-Mail: hwu2@dragon.nchu.edu.tw (H.-Y.W.); Telephone: +886-4-22840369; Fax: +886-4-22862073

Supplementary Figures

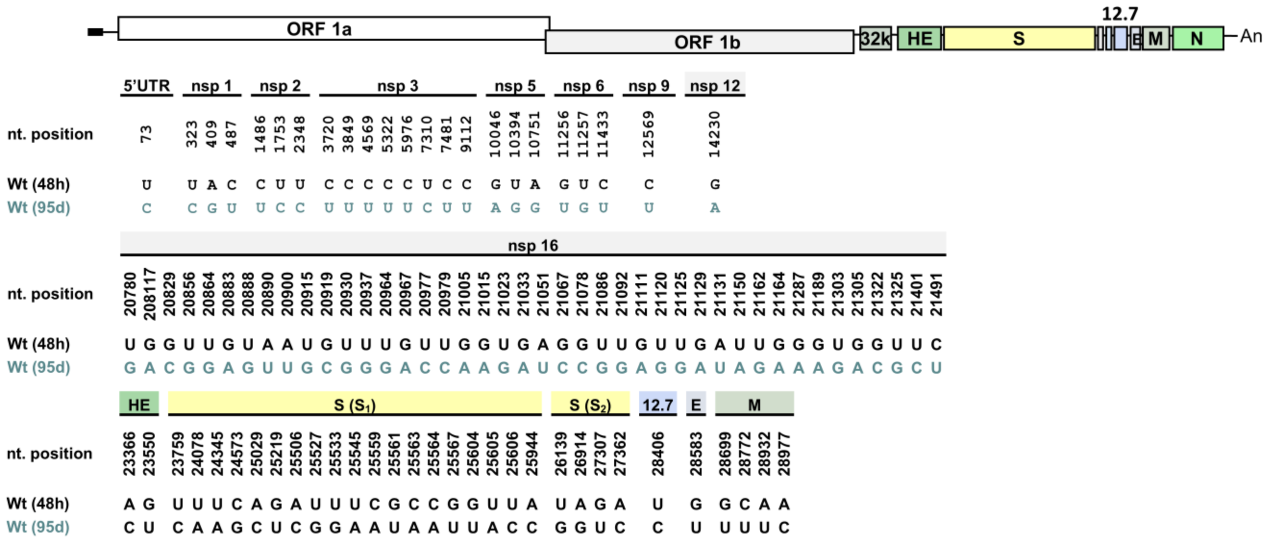

Figure S1. Linear schematic of BCoV genome showing the location of mutated nts under the selection pressures. Wt(48h): Viral RNA collected from fresh HRT-18 infected with wt BCoV at 48 hpi. Wt(95d): Viral RNA collected from HRT-18 cells after 95 d of persistent infection with wt BCoV. S_1: S_1 subunit, S_2: S_2 subunit.

A

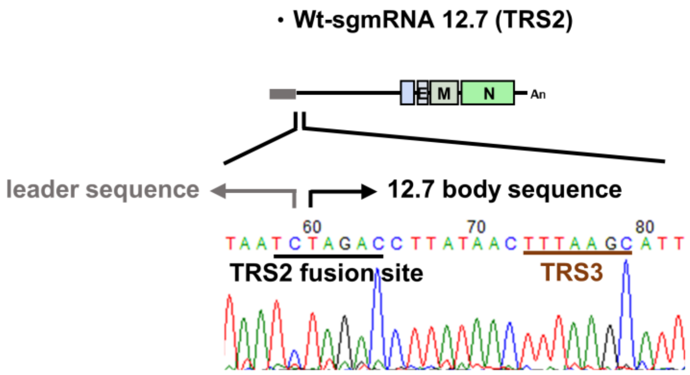

B

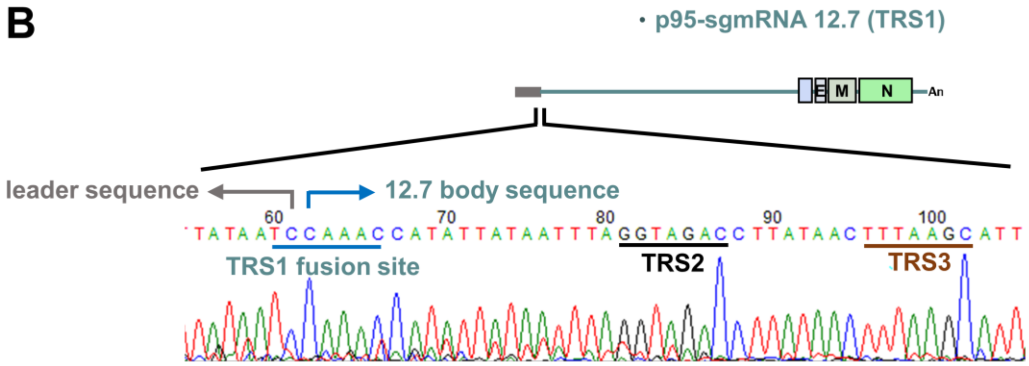

Figure S2. Sequencing analysis showing the leader-body fusion sites employed for synthesis of sgmRNA 12.7. (A) SgmRNA 12.7 synthesis (Wt-sgmRNA 12.7) with TRS2 as a leader-body fusion site in HRT-18 cells freshly infected with wt BCoV. **(B)** SgmRNA 12.7 synthesis (p95-sgmRNA 12.7) with TRS1 as a leader-body fusion site in HRT-18 cells with persistent BCoV infection (95 d).

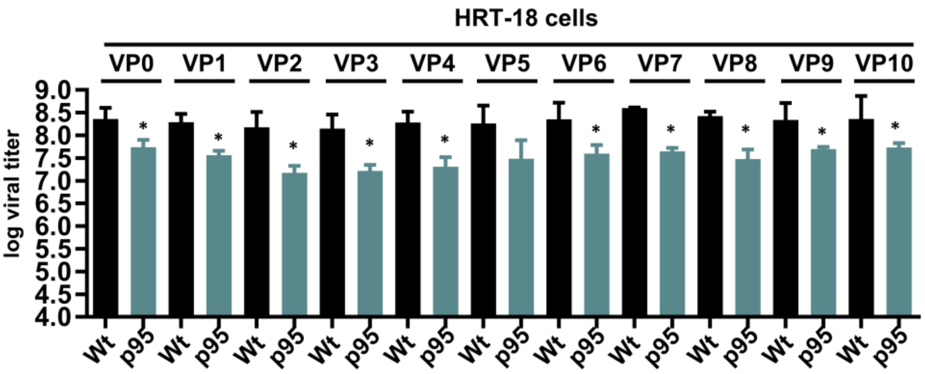

Figure S3. The virus titer of wt BCoV (Wt) and BCoV-p95 (p95) in fresh HRT-18 cells at VP0-VP10 as determined by the plaque assay. The values represent the mean ± standard deviation (SD) of three individual experiments. Statistical significance was evaluated using a *t*-test: *$P < 0.05$.

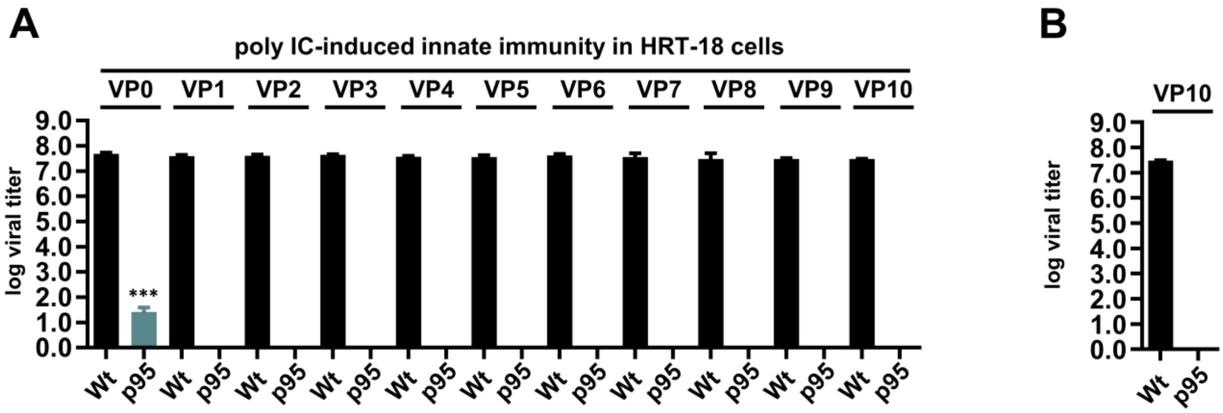

Figure S4. The virus titer of wt BCoV (Wt) and BCoV-p95 (p95) in fresh HRT-18 cells infected with Wt or p95 in the presence of poly IC at VP0-VP10 (A) and at VP10 (B) as determined by the plaque assay. The values represent the mean ± standard deviation (SD) of three individual experiments. Statistical significance was evaluated using a *t*-test: ***$P < 0.001$.

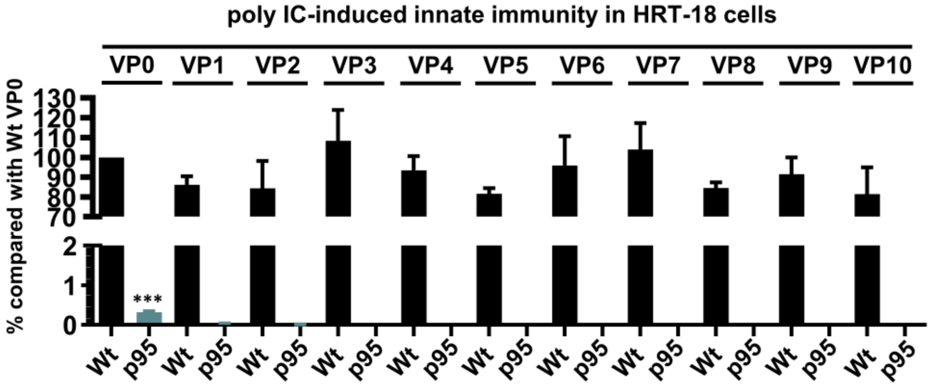

Figure S5. The relative amounts of genome between Wt and p95 from fresh HRT-18 cells infected with Wt or p95 in the presence of poly IC at VP0-VP10 as measured by RT-qPCR. The values represent the mean ± standard deviation (SD) of three individual experiments. Statistical significance was evaluated using a *t*-test: ***$P < 0.001$.

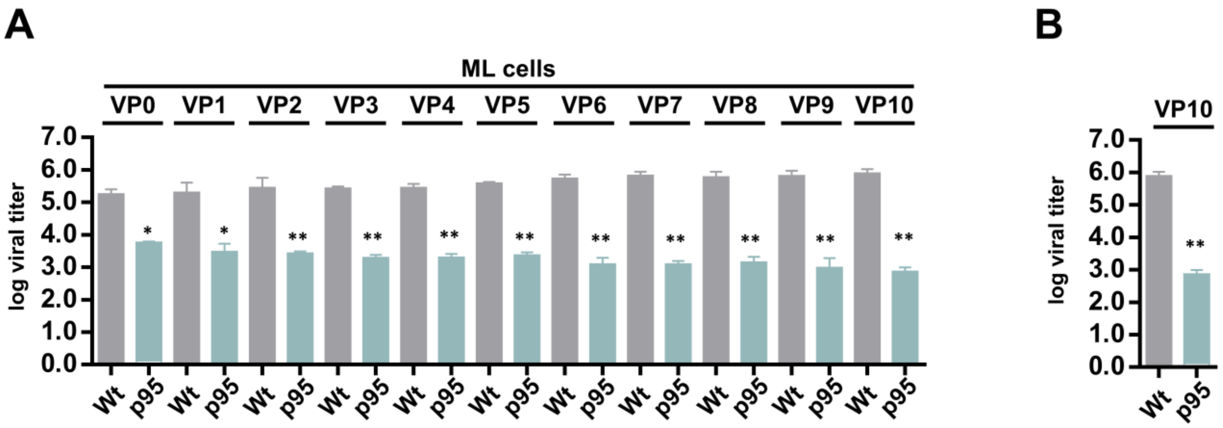

Figure S6. The virus titer of wt BCoV (Wt) and BCoV-p95 (p95) in fresh ML cells infected with Wt or p95 at VP0-VP10 (A) and at VP10 (B) as determined by the plaque assay. The values represent the mean ± standard deviation (SD) of three individual experiments. Statistical significance was evaluated using a *t*-test: *P < 0.05, **P < 0.01.
